# Line-field confocal optical coherence tomography of miliaria crystallina: An in vivo, three-dimensional imaging

**DOI:** 10.1016/j.jdcr.2024.03.014

**Published:** 2024-04-17

**Authors:** Anna Elisa Verzì, Giuseppe Micali, Francesco Lacarrubba

**Affiliations:** Dermatology Clinic, University of Catania, Catania, Italy

**Keywords:** imaging, LC-OCT, line-field confocal optical coherence tomography, miliaria crystallina

## Clinical presentation

**Fig 1:**
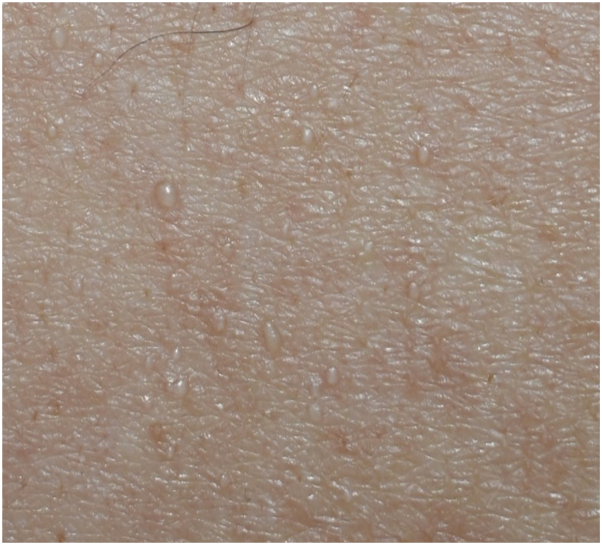
Small, clear, vesicles on the back of the patient.

**Fig 2:**
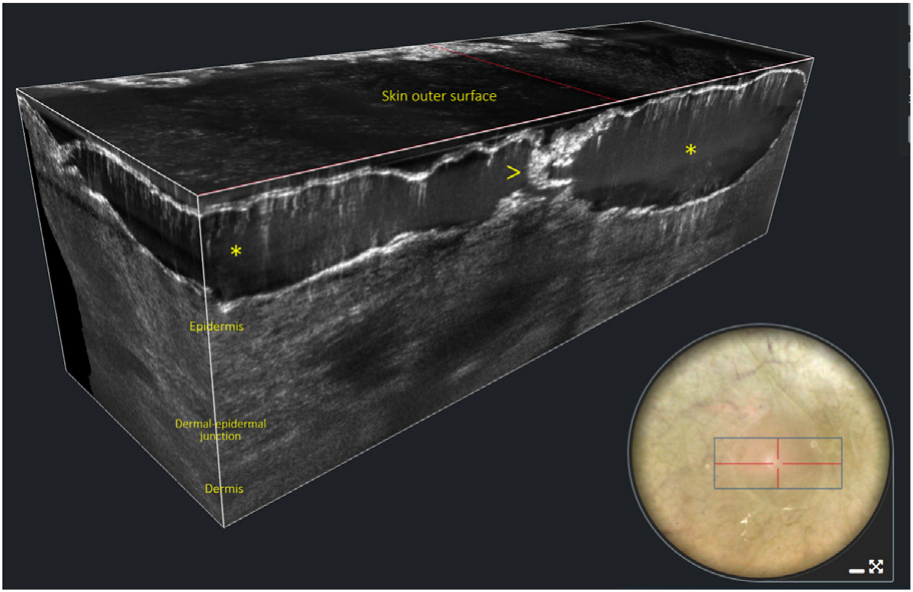
Three-dimensional line-field confocal optical coherence tomography image of a vesicle showing in the epidermis, just below the stratum corneum, the presence of roundish, hyporeflective, black areas (*asterisks*), corresponding to entrapped sweat, centered by a hyperreflective white, spiraliform structure (*arrow*), corresponding to the sweat duct. Insert: dermatoscopy of the vesicle showing a roundish, transparent structure.

A man in his 60s with a 3-week history of bed immobilization for major surgery, presented with several, small, clear, fragile, itching vesicles on his back (Fig 1). There was no erythema or umbilication of the lesions. Dermatoscopy of the vesicles was noncontributory showing roundish, transparent structures (Fig 2, insert). A clinical diagnosis of miliaria crystallina was suspected.

## Line-field confocal optical coherence tomography appearance

Line-field confocal optical coherence tomography (LC-OCT) examination of the vesicles confirmed the diagnosis of miliaria crystallina by showing in the epidermis, just below the stratum corneum, the presence of roundish, hyporeflective, black areas, corresponding to entrapped sweat, centered by a hyperreflective white, spiraliform structure, corresponding to the sweat duct (Fig 2). A simple patient mobilization was suggested, and the patient spontaneously healed after 3 days.

## Key message

Miliaria crystallina, or heat rash, or sudamina, is a common benign skin condition, triggered by high temperatures, and clinically characterized by multiple, tiny, itching vesicles of the trunk. It is caused by eccrine glands ductal obstruction at the level of the stratum corneum resulting in backflow of eccrine sweat and intraepidermal retention.^1^ Miliaria crystallina is generally observed in neonates and infants, but it may also occur in adults exposed to high heat and humidity, in case of febrile illnesses and/or bed immobilization. The upper portion of the trunk is mostly affected and, typically, there are no signs of inflammation. Differential diagnosis includes a series of rashes such as herpes simplex or varicella, bacterial or fungal folliculitis, neonatal acne, and drug reactions.^2^ Histopathologically, miliaria crystallina displays subcorneal or intracorneal vesicles from the intraepidermal portion of the duct.^2^ As spontaneous resolution generally occurs following temperature reduction, early recognition may avoid unnecessary skin biopsies and treatments.

LC-OCT is a new noninvasive tool for *in vivo*, real-time skin imaging. It uses a supercontinuum laser source (600-900 nm) and a line scan camera to measure the echo time delay and amplitude of light backscattered from cutaneous microstructures, providing *in vivo* high-resolution skin images in vertical and horizontal mode with a penetration depth of up to 500 μm.^3^^,^^4^ The software also provides 3-dimensional skin reconstructions. An integrated dermatoscopic camera allows the precise positioning over the area to be examined. LC-OCT allows a real-time microscopic evaluation of the different layers of the epidermis and dermis and their structures and findings with cellular level definition and demonstrated its usefulness for the diagnosis and treatment monitoring in a series of inflammatory, infectious, and neoplastic skin disorders.^3^^,^^4^

Limitations of LC-OCT, similar to other advanced imaging devices, such as reflectance confocal microscopy, include the equipment cost and nonportability. Best use is in hospital/research settings, and training for image interpretation is required.

In our case, LC-OCT was noninvasively able to enhance the clinical diagnosis of miliaria crystallina by showing in real time the underlying histopathologic alterations, in particular the subcorneal vesicles centered by the spiraliform sweat duct. In conclusion, although the diagnosis of miliaria crystallina is generally clinical, LC-OCT may be useful in doubtful cases, especially in children being noninvasive.

## Conflicts of interest

None disclosed.
